# Noncommunicable Disease and Health Care-Seeking Behavior Among Urban Camp-Dwelling Syrian Refugees in Lebanon: A Preliminary Investigation

**DOI:** 10.1089/heq.2020.0106

**Published:** 2021-04-30

**Authors:** Fatima M. Karaki, Ola Alani, Maya Tannoury, Farrah L. Ezzeddine, Robert E. Snyder, Arifi N. Waked, Zouhair Attieh

**Affiliations:** ^1^ Refugee and Asylum Seeker Health Initiative, Department of Medicine, University of California, San Francisco, San Francisco, California, USA.; ^2^ Institute for Global Health Sciences, University of California, San Francisco, San Francisco, California, USA.; ^3^ Faculty of Health Sciences, American University of Science and Technology, Beirut, Lebanon.; ^4^ Department of Epidemiology, Harvard T.H. Chan School of Public Health, Boston, Massachusetts, USA.

**Keywords:** refugee, Syria, health care, conflict, noncommunicable diseases, Lebanon

## Abstract

**Purpose:** Syrian refugees (SRs) in Lebanon are often relegated to informal camps with poor living conditions and substandard access to health care. This study examined the unique condition of urban camp-dwelling SRs in Lebanon. This population is rarely studied as they are marginalized and difficult to access. We sought to assess the prevalence of noncommunicable diseases (NCDs) and health care-seeking behaviors within this population.

**Methods:** A randomized group of urban camp-dwelling SR participants completed a survey on disease burden, health care-seeking patterns, and attitudes toward care. A second group completed interviews regarding health care experiences. We present descriptive population and epidemiologic measures to quantify NCD burden and health care-seeking behaviors.

**Results:** Of 101 participants, 39% reported arthritis, 25% skin infection, 24% high blood pressure, 16% chronic lung conditions, 16% eye disease, and 15% diabetes. Major themes from interviews with SRs included poor living conditions, high cost of health care services, and perceived discrimination by health care workers (HCWs). The major theme from interviews with HCWs was a perception that SRs received health care services similar to members of surrounding communities.

**Discussion:** In this population, we found a higher prevalence of NCDs at younger ages than in the general SR population. We described perceived barriers to accessing health care, including the noteworthy finding of perceived discrimination by HCWs in a linguistically and culturally related host community. We discussed social determinants of health within the camp and refugees' ability to access health care services.

## Introduction

In 2017 there were an estimated 25.4 million refugees worldwide, including more than 5 million Syrian refugees (SRs). The majority resettled in Lebanon, Jordan, and Turkey.^1^ In preconflict Syria, noncommunicable diseases (NCDs) were the leading cause of morbidity and mortality, accounting for 77% of all deaths. Following the onset of conflict, there have been serious and well-founded concerns related to the deteriorating health of refugees as a direct result of forced displacement.^2,3^

At the peak of the crisis, Lebanon hosted an estimated one million registered and half a million unregistered SRs. Because Lebanon is not a signatory to the 1951 United Nations (UN) Refugee Convention formally recognizing refugee rights, Syrians that flee to Lebanon are not formally defined as “refugees.” Instead, they are termed “displaced.”^4^ Thus, they cannot legally seek asylum or permanently resettle in the country.^5^ An estimated 75% of SRs in Lebanon lack formal residency status, limiting their ability to obtain health care, education, and employment.^6^ As a function of these inequalities, they are relatively understudied and their health concerns are poorly understood.

In response to the SR crisis, the UN implemented the Regional Refugee & Resilience Plan, aiming to assist SRs and host countries by providing support and strengthening existing systems.^7^ Participating nations' failure to meet funding targets set by the UN High Commissioner for Refugees (UNHCR) has severely impaired relief efforts, particularly in the health care sector.^8^ Registered SRs in Lebanon can obtain health care at UNHCR-affiliated health care facilities accessible through the “MediVisa” Lebanon insurance program, allowing registered refugees to pay a fee (approximately $2.00–3.33 US) for each medical consultation. UNHCR covers up to 75% of the overall cost and 85% of the cost of laboratory and diagnostic tests for children under 5 years and adults over 60 years. Registered refugees are only eligible for hospital admission in the event of a life-threatening emergency. Coverage varies based on severity of condition and vulnerability status. Unregistered refugees only receive UNHCR health services for antenatal and pediatric care. SR can also access care through private Lebanese health facilities, paying fees similar to uninsured Lebanese citizens.^9^

Among the limited studies examining SR's access to health care services in Lebanon, cost was described as the main barrier to obtaining care.^10–12^ The only nationwide survey to date reported that in over half of SR households in Lebanon, a member suffered from at least one of five common NCDs: hypertension, cardiovascular disease, diabetes, chronic respiratory disease, or arthritis. A 2015 survey of SRs in Lebanon found that the majority of households were not consistently able to afford medical care or prescription medication.^11^ Further research into the health care needs of SRs is needed as a 2019 scoping review found that only nine studies addressed NCDs in SRs in Lebanon. Of these, only four were original research articles and none examined urban camp-dwelling populations.^12^

Urban camps hosting refugees can be overcrowded with substandard housing and poor municipal infrastructure, placing residents at greater risk for both NCDs and communicable diseases.^13^ In 2016, many SRs in Beirut lived in the Bourj al-Barajneh Refugee Camp (BBRC), originally established as one of the few urban Palestinian refugee (PR) camps.^14^ As such, this SR population is in the rare situation of a displaced population hosted by a refugee population within a host country. This camp has also been classified by UNHCR as among the most impoverished and underserved areas in the country.^15,16^

We sought to assess NCD burden and explore barriers to health care access among urban-dwelling SRs living in BBRC, as perceived by both SRs and health care workers (HCWs). We also sought to elucidate the attitudes of SRs and HCWs toward the quality of their health and health care services.

## Methods

### Study site

BBRC in Beirut, Lebanon. The size of the camp is ∼0.6 mi^2^ and houses ∼31,000 people.^17^ Approximately 48% of the camp's residents are recent SRs.^16^

### Inclusion criteria

SRs age 18+ years who left Syria during or after 2011 residing in BBRC. Participation was limited to one individual per household. We also included HCWs employed at facilities used by SR participants, including Haifa Hospital, a Médecins Sans Frontières Clinic, Al-Kayan Primary Health Clinic, and Al-Shuly Pharmacy.

### Data collection

Participants were selected from a randomized list of all SRs registered with the camp's Popular Committee. Potential participants were selected using systematic random sampling and were invited to complete either a survey or interview. Surveys and interview guides were written in Arabic and had been pilot tested on native Arabic speakers, including Syrian citizens, to ensure cultural competence. All interviewers were native Arabic speakers. Exhaustive efforts were made to ensure shared linguistic and cultural norms between research participants and members of the research team. This allowed us to better ensure a dynamic, in which participants maintained agency in the decision to engage with researchers.

Four research team members administered a self-completed standardized survey to assess demographic characteristics, self-reported prevalence of NCDs, health care-seeking behaviors, barriers to care, and general attitudes regarding participants' health and health care services. Semistructured interviews were administered in accordance with the consolidated criteria for reporting qualitative research (COREQ) checklist by a member of the research team to a second group of participants.^18^ Interview guides were used to discuss participants' living conditions, self-reported medical history, and experiences accessing health care. HCWs discussed their experiences caring for SRs. Interviews were audiorecorded with permission. Hand-written notes were used when permission was not granted. Upon completion, all participants were given a meal voucher for a nearby restaurant.

### Data analysis

We summarized descriptive statistics without formal statistical analyses due to the relatively small sample size. A memoing technique was used to summarize each interview and identify main themes and concepts. Open coding followed by axial coding were used for the first few transcripts, synthesizing a codebook, which was used for the remaining transcripts. A framework table was used for data mapping and analysis.

### Ethics approval

This study was approved by the Institutional Review Boards at the University of California, San Francisco (UCSF), study number 16-21400, and the American University of Science and Technology (AUST), Lebanon, study number 201705-01. All participants provided written, informed consent. All interviewers underwent 2 days of in-person training and completed online Human Subjects Protection Training before conducting interviews.

## Results

### Survey results

Descriptive and demographic information can be found in Table 1. Self-reported prevalence of NCDs can be found in Table 2. UNHCR services provided to SRs and the percentage of participants who used these services can be found in Table 3. Participants reported having last seen a physician at a mean of 3.4 months before their interview (median: 1 month, range: 0–36 months). Of 95 participants who sought some form of care, 56% saw a specialist, 16% saw a primary care physician, 4% saw an emergency physician, and 23% saw a pharmacist or nonphysician HCW. Health care facilities included nongovernmental organizations (NGOs) or charity facilities (39%), private clinics (20%), pharmacies (18%), and hospitals (8.4%). Of the 58% of participants who reported being prescribed chronic medications, 42% reported being unable to obtain these medications. Following symptom onset, 42% of participants waited over 1 week before seeking medical care, including 19% who waited at least 3 months. Among these participants, 86% cited cost as the primary reason for this delay.

**Table 1.** Demographic and Household Characteristics of Syrian Refugees Living in Bourj-al-Barajneh Refugee Camp, Lebanon, 2017

	Surveyed participants	Interviewed participants
Demographics		
Participants (*n*)	101	20
Female gender, *n* (%)	88 (88)	17 (85)
Median age (minimum to maximum)	33 (19–61)	34 (24–67)
Formal education, %		
0–5 Years	29	—
5–10 Years	56	—
10 or more years	15	—
Marital status, %		
Married	85	85
Widowed	12	10
Not currently married	3	5
Years displaced from Syria, %		
0–1	6.7	10.5
1–2	10.7	15.8
3–4	47.1	26.3
5–6	35.6	47.4
Household characteristics		
No. of residents per household mean (standard deviation)	2.9	—
Children under 5 years old in home, %		
0	30	15
1–2	48	15
3 or more	23	70
Adults older than 65 years old in home, %		
0	77	—
1 or more	23	—
Pest presence around home, %		
Rats/mice	83	—
Mosquitoes	80	—
Cockroaches	81	—

**Table 2.** Self-Reported Prevalence of Select Communicable and Noncommunicable Diseases Among Syrian Refugees Living in Bourj-al-Barajneh Refugee Camp, Lebanon, 2017

Disease/condition	Self-reported prevalence (%)
Heart disease	6
Neurologic disease (dementia, Parkinson's, or epilepsy)	8
Kidney disease	12
High cholesterol	14
Diabetes	15
Glaucoma or cataracts	16
Chronic lung disease (COPD, emphysema, or asthma)	16
High blood pressure	24
Skin infection	25
Arthritis	39

COPD, chronic obstructive pulmonary disease.

**Table 3.** United Nations High Commissioner for Refugees Services and Benefits Received by Interviewed Participants in Bourj-al-Barajneh Refugee Camp

UNHCR services/benefits of interviewed participants		
Registration status, %		
Registered	—	95
Unknown	—	5
Health insurance, %		
Yes	—	70
No	—	20
Unknown	—	15
Food subsidies, %		
Yes	—	35
No	—	35
Unknown	—	30
Rent assistance, %		
No	—	90
Unknown	—	10
Heating during winter, %		
Yes	—	20
No	—	45
Unknown	—	30

UNHCR, United Nations High Commissioner for Refugees.

Participants were asked about their beliefs regarding the most important health concerns facing SRs. Greatest concerns were related to mental illness (47%), pediatric illness (39%), communicable disease (32%), NCDs (19%), and reproductive health (13%). Only 42% of participants believed SRs benefited from the health care that was offered by NGOs, UNHCR services, or charities.

### Interviews

This portion of the study included 20 SR participants and 4 male HCW, employed as either a nurse, nurse administrator, pharmacy owner, or physician administrator. Only four HCWs participated due to general reluctance among HCWs approached to grant interviews. Themes included disease burden, health care access, and living conditions, described in Table 4.

**Table 4.** Summary of Interviews with Syrian Refugees and Health Care Workers in Bourj al-Barajneh Refugee Camp

Theme	Summary	Representative quotes/anecdotes
Disease burden	Most SRs reported at least one household member suffering from an NCD, particularly hypertension, diabetes, or back pain; child-related illness; or pregnancy-related illness. Some reported mental health issues, including stress, anxiety, or depression. Most HCWs believed that maternal and pediatric health issues were more common in SR than the general population. Some SRs and two HCWs cited the camp environment and their living conditions as the main source of stress, depression, or children's illnesses, (e.g., asthma, chronic fever, or vomiting).	“Depression. I'm 32 years old, but honestly what I've experienced in the last five years, I have never seen my entire life [crying]. Living under pressure, what do you do? Laugh? Especially when you have children, they will feel the pressure and soon will live it too.” (32 F, SR with five children) A 27-year-old SR reported that three of her four children developed constant diarrhea and vomiting shortly after arriving in the camp.
Health care access		
(1) Cost considerations	The costs of health care, particularly for diagnostic testing, medications, or emergency care, were described as prohibitive by most participants. Some reported that the discounted copay of $3 remained excessive. Due to cost, some did not seek further treatment (e.g., obtaining medications), some consulted pharmacies, and a few even returned to Syria. Most HCWs believed that health services were adequate and that costs were low compared with what was paid by Lebanese citizens.	“My husband makes $20 a day which has to pay for transportation, rent, food, and water…everything, clothes and what-not.” (30 F, SR with four children) One SR stated the cost to remove a plaster cast from her son's leg was $43, so she did it herself at home using a knife. “The hospital wouldn't help me deliver until we pay. My husband begged them till they let me in, and mind you, my baby was almost out.” (32 F, SR with five children)
(2) HCW attitudes	Some SRs cited negative treatment by certain HCWs at specific health facilities as a factor that influenced their access to care. Some described their own negative interactions, while others witnessed or heard of these incidents secondhand. In all cases SRs decided not to return to the facility. Three HCWs stated that SRs had an inability to follow instructions, lack of education, and/or poor hygiene leading to negative effects on their health. HCWs listed a variety of other challenges such as facilities exceeding their capacity and lack of funding. HCWs felt that health care challenges were not unique to SRs, and were similar to those faced by Lebanese citizens.	One participant stated that during labor, the physician “shushed” her and told her she would need to “get out” and deliver the baby on her own if she continued to groan. The participant went on to describe how HCW were “bullies” who felt “burdened by us [Syrians].” “I went to a doctor and he was making fun of me … I went again, the same doctor asked how I was—looking at me condescendingly … My friend came after me -and she's a bit overweight, he asked her ‘are you gaining weight rapidly?’ she said, ‘yes.’ He told her, ‘okay then shut it’ … my friend said, ‘shut what?’ ‘your mouth,’ he said … [this happens] just because we're paying through UNHCR, but if you went to any [private] clinic and paid out of pocket, they will salute you. But just like that, we're destined for humiliation.” (36 F, SR) “… if someone wants to shower they can use a bottle of water, it's not a problem … All of us [in Lebanon] suffer from water issues … You instruct the mother to clean, wipe, and dry [her child]. The child comes back all messy … She changes the diaper, but she puts it on top of [the feces].” (Male HCW, nurse) “You get a 35-year-old male with ten children, half of them were born in Lebanon!” (Male HCW, nurse)
(3) Health care services	Some SRs reported that their access was impacted by poor quality of care, overcrowded facilities, lack of information regarding available services, and distance to the facilities, many of which were located outside the camp.	“The problem is how do you get inside [the clinic]? … inside it's overcrowded and disorganized, you'd sit for a whole day waiting with no cooking, no chores done at home! … Why not bring [services] to the camp?” (36 F, SR) One SR noted she needed to borrow money to cover transportation costs to a clinic in a camp ∼4 km away.
Living conditions and organizational support	Most SRs raised concerns about living conditions in the camp, including a lack of clean water and garbage collection, high unemployment, and high rent. Some SRs described constant harassment and negative treatment by Palestinians in the camp, and by Lebanese outside of the camp. Some SRs discussed UNHCR benefits such as health coverage, rent support, a nutrition card for food subsidies, and subsidized heating in the winter. A few SRs did not know whether they had UNHCR health coverage or that their health care was subsidized by UNHCR. Some SRs had nutrition cards, while among others it was discontinued or never offered. Most indicated that UNHCR services were inadequate, and a few described unfair treatment. Most HCWs believed SRs received adequate help from the UNHCR. Some recommendations from HCWs included increased funding, and one HCW suggested understanding the needs of the community and educating SRs about what was available, before implementing futile intervention programs.	“You'd have to walk on a mountain of garbage to get [to the house].” (36 F, SR with six children) “Their treatment of us is not good … they drop words, they criticize you ‘Syrians are dirty’ ‘where did Syrians come from’ … nothing pleases them.” (35 F, SR pregnant with six children) “Some Syrians in the camp are receiving everything [benefits]. UNHCR has a lot of unfairness, a lot. They give to people who don't need help and leave those who are in need. Yesterday I went to the ‘discounted market’ and saw people… [who] don't need help … however, those who go to get oil, sugar, and rice and feed their children they [UNHCR] cut them off.” (37 F, SR with 3 children) “UNHCR has clinics and dispensaries for them [Syrians] whether it's women's, primary health, pediatrics and all, [Syrians] are getting treated.” (HCW, Male pharmacy owner) “…if you provide [for a] real need—and you don't create a need to just open a project—then people will [come] … and make sure to provide education and promotion about the services that you [already] have.” (HCW, Male nurse administrator nurse)

F, female; HCWs, health care workers; NCD, noncommunicable disease; SRs, Syrian refugees

### Disease burden

SRs reported NCDs such as hypertension, diabetes, asthma, and back pain; mental health issues (e.g., stress, anxiety, and depression); pediatric illnesses; injuries; and pregnancy-related concerns. Some cited their environment and life in the camp as the main factors contributing to their illness.

HCW's agreed that NCDs are common among SRs; however, they believed rates were similar to Lebanese and Palestinian populations living in and around BBRC. HCWs noted that they mostly treat seasonal, pediatric illnesses, and maternal health issues in SRs. Some HCWs attributed this to SR's low socioeconomic status and overcrowded living conditions.

### Health care access

All SRs noted high cost of medical care and resultant increase in financial burden. Costs included physician fees, diagnostic testing, and medications. For some SRs, UNHCR partially or fully financially covered physician visits but not diagnostic tests or medications. These could cost several times more than the visit itself. Almost all SRs said that they would go to the pharmacy when sick to avoid physician fees. Several mentioned they would go to other health care facilities since they could not afford a physician. Some said they had not consulted a physician, done necessary tests, or bought necessary medication due to cost.

All HCWs believed that SRs received sufficient medical assistance through UNHCR. One HCW stated that their facility assists SRs who are unable to pay by forgiving fees, reducing charges, or giving patients a grace period. Another HCW said they sometimes pay out of pocket themselves to cover SR fees.

### HCW attitudes

Some SRs experienced humiliation from HCWs firsthand, witnessed it toward another SR, or heard of others' negative experiences. This deterred them from visiting a health care facility. Two HCWs from different facilities believed SRs had issues with personal hygiene and education level.

### Health care services

Several SRs found distance to health care facilities and wait times at medical facilities to be a barrier to treatment. Two interviewed SRs believed that going to a pharmacy was equivalent to seeing a doctor as both prescribe the same medication. Some also did not know available health services and whether they received financial coverage.

### Camp environment

Most SRs described crowded living conditions with families of five or more living in one to two rooms. SRs described surrounding areas as dirty, with piles of uncollected garbage, the smell of sewage, and rodent and cockroach infestations.

## Discussion

We report results from a survey of the urban camp-dwelling SR population in BBRC in Beirut, Lebanon. Similar to SRs in rural areas of Lebanon and Jordan, SRs in the urban BBRC live in overcrowded and unhygienic conditions with an average of 6.8 people per home.^19,20^ In this population, we found a high prevalence of NCDs, as described in Table 3. As described in Table 4, we found that perceived discrimination by HCWs was cited as a barrier to health care access. However, health care-associated costs were cited as the main barrier to accessing care. Participants reported that the most pressing health concerns facing their community were mental illness, pediatric illness, and communicable diseases.

The prevalence of NCDs in this population was higher than the most frequently cited nationwide study of NCDs among SRs in Lebanon: arthritis (39% in participants vs. 7.9% nationwide), hypertension (24% vs. 7.4%), lung disease (16% vs. 3.8%), and diabetes (15% vs. 3.3%).^10^ Our finding of hypertension in 24% of participants was similar to prior studies of residents of preconflict Syria, ranging from 24.9% to 29.5%. However, our reported 15% prevalence of diabetes was higher than the 8.8% diabetes prevalence reported in preconflict Syria.^10,21,22^

Our study is among the first to report a relatively high burden of skin and eye diseases, conditions which are understudied in most refugee populations. Targeted research is needed to identify specific etiologies of these complaints.

Among participants who reported needing medication to treat an NCD, 42% were not able to obtain it regularly due to cost, leaving their disease untreated. Cost issues were likely compounded by recent laws enacted by the Lebanese government that have made it extremely difficult for refugees to work and operate businesses in Lebanon.^23^

Although several organizations subsidize SR health care in Lebanon, 58% of participants who sought care paid fully out of pocket. This gap between organizational policy and the lived reality for SRs may be due to incomplete knowledge of organizational support, such as where to seek care or what services are available. Previous studies have shown that SRs were unable to access care, leading them to feel that health care was random, unfair, or insufficient.^24^ This is in part due to refugees being treated as recipients rather than stakeholders in their own health and wellbeing.^25^ In contrast, HCWs interviewed generally felt that services provided were both adequate and cost appropriate. This disconnect is a commonly described tension in many host communities.^26^

When seeking care, the majority of participants saw a specialist rather than a primary care physician. As the majority paid out of pocket for health care services, they may have sought to skip primary care fees in favor of direct access to a specialist. Participants also expressed that seeing a pharmacist in lieu of a physician could yield the same result, a prescription, for less money. However, this can lead to numerous problems, including increasing antibiotic resistance rates due to improperly treated infections, poor health outcomes due to incorrect diagnoses, and other adverse consequences.^13,27^ Some SRs also visited informal SR HCWs, as has been described in other regions of Lebanon.^19,28^

As described in Table 4, a notable finding of this study is that participants sometimes avoided care due to perceived discrimination by HCWs. Participants frequently cited poor treatment by HCWs because of their ethnicity, socioeconomic status, or receipt of subsidized services. This was further reinforced by participating HCWs, who described refugees as having poor hygiene, low health literacy, and entitlement to care. While this phenomenon had not been described in the literature at the time of the study, two recent reports note HCW discrimination against SRs, although in different contexts from our findings: Honein-AbouHaidar et al.^28^ note Lebanese host community HCW discrimination against SR informal HCW, and Nabulsi et al.^19^ observed that rural SRs reported HCW discrimination in the Bekaa valley. These reports signal a potentially more widespread pattern of discrimination that should be explored. Perceived health care discrimination against refugees has been previously documented,^29,30^ but most of these studies were conducted in countries with broader differences (e.g., language/culture) between host and refugee communities. Our finding is noteworthy given the shared culture and language in the region.

This may reflect recent findings concerning the pervasiveness of antirefugee sentiment in Lebanon.^31^ Some antirefugee sentiment may be attributable to preexisting tensions in Lebanon as a result of the ongoing economic crisis. Competition for employment, housing, and resources is fierce. An early poll noted that the majority of Lebanese interviewed believed that SRs threaten national security and stability, and about 50% thought that SRs benefit unfairly from financial aid.^3^ Some Lebanese also believe that it is now safe for refugees to return to Syria due to recent forced deportations of over 2,500 SRs along with the voluntary repatriation of 170,000 more.^32^ Moreover, BBRC provides a unique camp setting in which one refugee population (PR) hosts another (SR). In spite of shared language, religion, and political views, tension was still noted between the two communities both informally by the research team while conducting the study, and in a prior report.^33^ PRs expressed frustration that SRs received attention and funding from international donors that dwarfed support of PRs. HCWs in our study expressed the belief that PRs and Lebanese citizens in the country facing similar problems did not receive the same support as SRs. A valuable finding of this study is this rift that competition for limited economic and health care resources can create, even in deeply similar communities.

In conducting our research, we strived to build relationships and amplify refugee participants' opinions, engaging them as equals. However, there are some limitations of our study that may inhibit the generalizability of results. We recruited a relatively homogenous participant group of younger women who self-reported their illnesses without medical confirmation. The lack of older participants in our study may have been due to the younger camp demographic, problems related to transportation to the project site, or self-selection regarding where participants had settled.^34^ Furthermore, the list of refugees provided may not have included all SRs in the camp. Conducting the study alongside the camp's Popular Committee authority may also have limited some participant responses due to the power dynamics between this leadership and refugees. However, access to this population would not have been possible without the Popular Committee's explicit approval and endorsement. These factors may have contributed to conservative biases, in which we failed to include more vulnerable populations. Additionally, HCW participants in this study account for a small number of those serving SRs. This did not allow us to consider data saturation.

More robust efforts must be made to survey urban camp-dwelling refugee populations, representing some of the most vulnerable and understudied refugee groups. Those who settle in rural refugee camps may be more easily studied and better understood; however, both these and urban camp-dwelling refugees likely suffer deleterious health effects due to the numerous health inequities they encounter. More work must be done to understand differences in the characteristics of refugees who disperse among urban neighborhoods compared with those who settle in formal camps. Future studies should also focus on directly comparing subpopulations in and around these camps to better quantify health disparities. Future research should examine correlations between specific living conditions and increased prevalence of NCDs. Additionally, potential future interventions should focus on mechanisms to decrease cost and improve relationships between refugees and HCW to improve refugee access to care.

After establishing a sufficient evidence base regarding disease prevalence and needs assessment, focus can be shifted to evaluating interventions and effective public health prevention measures. Understanding how different programs operate within the camp, critically evaluating performance, and systematically incorporating effective care models into other areas will lead to long-term, evidence-driven interventions that will improve clinical outcomes among refugees.

## Ethics Approval and Consent to Participate

The study was approved by the Institutional Review Boards at the UCSF study number 16-21400 and the AUST, Lebanon study number 201705-01. All participants provided written, informed consent.

## Availability of Data and Materials

All relevant quantitative data are within the article and its Supporting Information files. Qualitative data cannot be shared publicly due to privacy concerns, as public availability would compromise patient confidentiality and participants did not consent to have their full transcripts or recordings made publicly available. Transcript excerpts underlying the qualitative results presented in the study are available upon request by contacting the corresponding author at fatima.karaki@ucsf.edu

## Abbreviations Used

AUST: American University of Science and Technology
BBRC: Bourj al-Barajneh Refugee Camp
COPD: chronic obstructive pulmonary disease
F: female
HCW: health care worker
NCD: noncommunicable disease
NGO: nongovernmental organization
PR: Palestinian refugee
SR: Syrian refugee
UCSF: University of California, San Francisco
UN: United Nations
UNHCR: United Nations High Commissioner for Refugees
